# Japanese Cohort Study Identifies an Association Between the Serotonin Transporter–Linked Polymorphic Region and Suicide Death

**DOI:** 10.1002/npr2.70120

**Published:** 2026-04-12

**Authors:** Shohei Okada, Toshiyuki Shirai, Masao Miyachi, Gentaro Yamasaki, Kiriko Minami, Ryo Tsukamoto, Kentaro Mouri, Takaki Tanifuji, Ryuichi Katada, Satoshi Okazaki, Ikuo Otsuka, Akitoyo Hishimoto

**Affiliations:** ^1^ Department of Psychiatry Kobe University Graduate School of Medicine Kobe Japan; ^2^ Division of Legal Medicine, Department of Community Medicine and Social Health Science Kobe University Graduate School of Medicine Kobe Japan; ^3^ Department of Rehabilitation Science Kobe University Graduate School of Health Sciences Kobe Japan; ^4^ Kobe University Inclusive Campus & Healthcare Center Kobe Japan

**Keywords:** 5‐HTTLPR, meta‐analysis, suicide

## Abstract

**Introduction:**

More than 720 000 individuals die by suicide annually worldwide, making it a major public health concern and a leading cause of death among young people, particularly in Asian populations. Dysregulation of the 5‐hydroxytryptamine system has been implicated in suicide risk. The *SLC6A4* gene encodes the serotonin transporter, which regulates synaptic 5‐hydroxytryptamine levels. Its promoter region contains a 44‐base‐pair insertion/deletion polymorphism, known as the serotonin‐transporter‐linked polymorphic region (5‐HTTLPR). We examined the association between the 5‐HTTLPR polymorphism and suicide death in a Japanese cohort, representing the largest sample from an Asian population to date.

**Methods:**

We included 448 suicide decedents and 361 controls. Genomic DNA was extracted from peripheral blood, and 5‐HTTLPR genotypes were determined using PCR. We evaluated genotype‐ and allele‐level associations with suicide. Additionally, we conducted a meta‐analysis incorporating previous studies.

**Results:**

The distributions of 5‐HTTLPR genotypes and alleles differed significantly between suicide decedents and controls (S allele: OR = 1.62, 95% CI = 1.26–2.09, *p* < 0.001). The associations remained significant in subgroup analyses by sex, age group, and violent methods. Age‐ and sex‐matched analyses yielded consistent results. However, the random‐effects meta‐analysis did not demonstrate a significant association.

**Conclusion:**

Our findings indicate a potential association between 5‐HTTLPR polymorphisms and suicide death in a Japanese population. Although further research in larger and diverse samples is warranted, these results may contribute to a better understanding of the biological underpinnings of suicide risk.

## Abstract 2

The odds ratios and corresponding 95% confidence intervals for suicide death by 5‐HTTLPR S allele, in main and subgroup analyses. Statistical significance is indicated.

## Graphical abstract

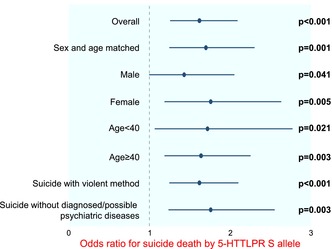

## 1 Introduction

According to the World Health Organization, more than 720 000 individuals die by suicide worldwide each year, representing a major public health concern and one of the leading causes of death among young people [1]. Notably, approximately 60% of suicide deaths occur in Asian countries, including Japan [2, 3]. In Japan, suicide is the first leading cause of death among individuals aged 10–39 years, and it also remains relatively common among those aged 40 years and older [4]. Twin, family, and adoption studies have demonstrated that suicide has substantial heritable components [5]. However, because of the difficulty of obtaining postmortem samples from individuals who have died by suicide, the genetic understanding of suicide has lagged behind that of many other psychiatric diseases and conditions.

Neurobiological research has suggested a role for 5‐hydroxytryptamine (5‐HT) dysfunction in suicide. Lower concentrations of 5‐hydroxyindoleacetic acid, a major 5‐HT metabolite, in cerebrospinal fluid have been observed in individuals with suicide attempts, and this reduction has been linked to a 4.6‐fold increase in suicide risk [6]. Monoamine oxidase A, encoded by the *MAOA* gene, is a mitochondrial enzyme responsible for the 5‐HT degradation, and genetic variants in this gene have also been associated with suicidal behavior [7].

The *SLC6A4* gene encodes the 5‐HT transporter (5‐HTT), which regulates synaptic 5‐HT levels by reuptaking the neurotransmitter into presynaptic neurons [8]. A 44‐base pair insertion/deletion polymorphism—known as the serotonin‐transporter‐linked polymorphic region (5‐HTTLPR)—is located in the promoter region of SLC6A4 and produces long (L) and short (S) alleles [9]. The L allele is associated with increased 5‐HTT expression, whereas the S allele has been reported to reduce the transcription activity of *SLC6A4* [10]. Although several previous studies have examined the association between 5‐HTTLPR polymorphisms and suicide, most have been conducted in European or North American populations, and the findings have been inconsistent [11, 12, 13, 14]. Given the substantial burden of suicide in Asia, replication studies with sufficiently large sample sizes in Asian populations are needed. In this study, we investigated the association between 5‐HTTLPR polymorphisms and suicide death in the largest cohort of Asian individuals assessed to date, consisting primarily of Japanese participants.

## 2 Methods and Materials

### 2.1 Participants

We included 448 suicide decedents and 361 healthy controls in this study, all of whom were of Japanese descent. Suicide death was confirmed through medicolegal examinations and police investigations in accordance with Japanese law. Autopsies of the suicide decedents were conducted at the Department of Legal Medicine, Kobe University Graduate School of Medicine. To obtain background information on the suicide decedents, including demographic characteristics and circumstances surrounding death, we used the data gathered by staff at the Medical Examiner's Office of Hyogo Prefecture and the Division of Legal Medicine at Kobe University.

The methods of suicide were categorized based on official reports and included jumping in front of a vehicle, jumping from heights, self‐inflicted penetrating wounds, drug overdose, taking poison, neck hanging, self‐burning, drowning, gas suffocation, other methods, and unknown. We subdivided suicide decedents into violent suicide (jumping from heights, jumping in front of a vehicle, self‐inflicted penetrating wounds, neck hanging, self‐burning, drowning, and gas suffocation) and nonviolent suicide (drug overdose and taking poison), in accordance with Dumais et al. [15].

Control participants were screened by at least two psychiatrists who evaluated them with unstructured interviews. The inclusion criteria of controls required the absence of any current or past psychiatric disorders, substance use disorders (excluding nicotine dependence), and family history of psychiatric disorders in first‐degree relatives.

### 2.2 Genotyping

Peripheral blood samples were obtained from all participants and stored at −80°C until analysis. Genomic DNA was extracted using the QIAamp DNA Blood Midi Kit (Qiagen, Valencia, CA, USA), and DNA concentration and purity were assessed with a NanoDrop spectrophotometer (Thermo Fisher Scientific, Wilmington, DE, USA). For all samples, polymerase chain reactions (PCR) were performed to determine the L or S alleles of the 5‐HTTLPR polymorphism. Each PCR reaction (10 μL total volume) contained 10 ng of genomic DNA, 5 μL of AmpliTaq Gold 360 Master Mix (Thermo Fisher Scientific), 10% GC enhancer, 0.1 μM of each primer, and 5 pmol of both forward and reverse primers.

Based on the protocol described by Hu et al., we used forward primer: 5′‐FAM‐GGCGTTGCCGCTCTGAATGC3′ and reverse primer: 5′‐GAGGGACTGAGCTGGACAACCAC‐3′ [16]. PCR amplification was performed under the following cycling conditions: an initial denaturation at 95°C for 10 min, followed by 40 cycles of 95°C for 30 s, touchdown annealing from 63°C to 55°C for 30 s, and 72°C for 30 s. A final extension was conducted at 72°C for 7 min. PCR products were analyzed using the SeqStudio Genetic Analyzer (Applied Biosystems, Waltham, MA, USA) and the GeneMapper Software version 6 (Applied Biosystems).

The expected amplicon sizes for the 5‐HTTLPR polymorphism were 529 base pairs (L allele) and 486 base pairs (S allele), consistent with previous studies [16]. Based on allele patterns, individuals were classified into three genotype groups: SS, SL, and LL.

### 2.3 Statistical Analysis

Statistical analyses were conducted using the R version 4.5.0 (R Development Core Team, Vienna, Austria). Difference in sex between suicide decedents and controls were evaluated using Fisher's exact test, and differences in age were assessed using Welch's *t*‐test among participants with available age information. The association between 5‐HTTLPR genotype (SS, SL, and LL) and suicide death was first examined using Cochrane–Armitage trend test. In addition, allele‐based associations (S vs. L) were analyzed using Fisher's exact test. Additionally, to account for potential differences in sex or age between the two groups, we conducted sex‐ and age‐matched analyses using EZR (R version 4.2.2).

As subgroup analyses, we performed the same tests stratified by sex and age group (under 40 vs. 40 and older). Participants without age information (13 suicide decedents and six controls) were excluded from the age‐related subgroup analyses. We further conducted subgroup analyses among controls and suicide decedents with violent methods (414 individuals whose methods we have confirmed). Furthermore, we conducted a subgroup analysis using a cohort comprising suicide decedents without possible or diagnosed psychiatric diseases and controls. For multiple testing, we applied the Benjamini–Hochberg method for correction. Statistical significance was set at *p* < 0.05 (two‐tailed).

Hardy–Weinberg equilibrium for genotype distributions was assessed using Haldane's exact test. Statistical power was estimated using Cohen's *h* effect size. To examine whether the observed association was independent of potential confounding factors, age‐ and sex‐adjusted logistic regression analyses were also performed, with genotype as the explanatory variable and phenotype as the objective variable. Dummy variables were coded as follows: sex (female = 1, male = 0), phenotype (suicide decedent = 1, control = 0), genotype (SS = 2, SL = 1, LL = 0), and allele (S = 1, L = 0).

### 2.4 Meta‐Analysis

To conduct meta‐analysis, we searched for candidate articles in PubMed through August 4, 2025, using the following search terms: (“serotonin transporter” OR “SERTPR” OR “5‐HTTLPR” OR “SLC6A4”) AND (“suicide”). This meta‐analysis was exploratory and was not preregistered. For this analysis, we considered previously published studies based on the following criteria: (1) examining the 5‐HTTLPR polymorphism in both controls and suicide decedents, (2) independent study from other included studies, (3) adequate and sufficient information for the data synthesis, and (4) language was limited to English. We first screened titles and abstracts, followed by full‐text assessment when necessary, and excluded studies that did not meet the eligibility criteria. Two reviewers (S.O. and T.S.) independently screened all records, and disagreements were resolved through discussion; if agreement could not be reached, a third reviewer S.O mediated the decision.

Phenotypes were categorized into suicide decedents and healthy controls. The meta‐analysis was conducted with the R package metafor, applying a random‐effects model to evaluate the association between 5‐HTTLPR alleles (S or L) and suicide risk, expressed as ORs. Statistical heterogeneity was assessed using the *I* ^2^ statistic, interpreted as follows: 0%–40%, might not be important; 30%–60%, may present moderate heterogeneity; 50%–90%, may present substantial heterogeneity; 75%–100%, be considerable heterogeneity [17].

## 3 Results

### 3.1 Genotype and Allele Analyses

Significant differences were observed in sex (*p* < 0.001) and age (*p* = 0.020) between suicide decedents and controls (Table 1). Detailed data of the participants were listed in Table S1. Overall, genotype analyses demonstrated a significant association between 5‐HTTLPR polymorphism and suicide death (*p* < 0.001). In addition, the S allele was significantly associated with suicide risk (OR = 1.62, 95% CI = 1.26–2.09; *p* < 0.001). After matching for age and sex, the cohort included 290 controls and 290 suicide decedents. In this matched cohort, both genotype (*p* = 0.001) and allelic analyses (OR = 1.70, 95% CI = 1.25–2.30; ***p* = 0.001**) demonstrated significant associations.

**TABLE 1:** Demographic and clinical characteristics.

Overall	Controls	Suicide decedents	*p*
Number	361	448	
Male/Female	161/200	299/149	< 0.001^a^
Age, mean ± SD	53.5 ± 19.2	50.4 ± 17.8	0.020^b^

Sex and age matched^c^	Controls	Suicide decedents	p
Number	290	290	
Male/Female	160/130	160/130	1^a^
Age, mean ± SD	50.1 ± 19.1	50.1 ± 19.1	0.981^b^

Abbreviation: SD, standard deviation.

^a^ *p*‐value was calculated using Fisher's exact test.

^b^ *p*‐value was calculated using Welch's *t*‐test.

^c^ We matched the sex and age of controls and suicide decedents with EZR (R version 4.2.2).

**FIGURE 1:**
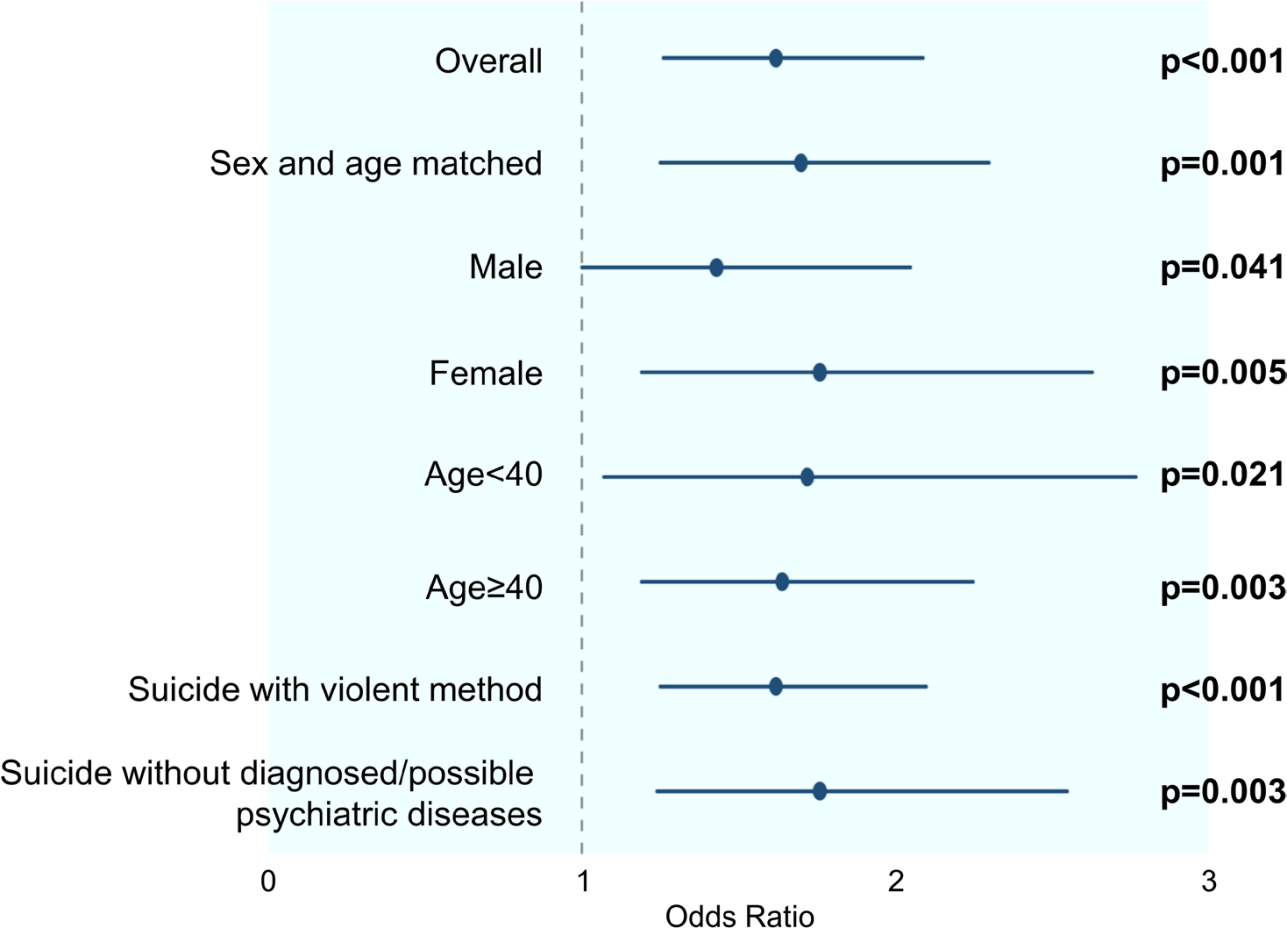
Odds ratios in the overall and subgroup analyses. Blue dots and lines indicate the odds ratios and corresponding 95% confidence intervals. The *p*‐values were adjusted with Benjamini–Hochberg methods.

In sex‐stratified analyses, the S allele remained significantly associated with suicide death in both males (OR = 1.43, 95% CI = 1.00–2.05; *p* = 0.041) and females (OR = 1.76, 95% CI = 1.19–2.63; *p* = 0.005). Similarly, significant associations were observed among individuals younger than 40 years (OR = 1.72, 95% CI = 1.07–2.77; *p* = 0.021) as well as those aged 40 years and older (OR = 1.64, 95% CI = 1.19–2.25; *p* = 0.003). Among controls and suicide decedents with violent methods, the association was also significant (OR = 1.62, 95% CI = 1.25–2.10; *p* < 0.001). Among controls and suicide decedents without possible or diagnosed psychiatric diseases, the association was also significant (OR = 1.76, 95% CI = 1.24–2.55; *p* = 0.003) (Table 2 and Figure 1).

**TABLE 2:** 5‐HTTLPR genotype and allele frequency of patients with suicide decedents and controls in this study.

	*n*	HWE^a^	Genotype S/S				Allele S				OR (95% CI)^d^	Power^e^
			S/S	S/L	L/L	*p* ^b^	Allele S	L	MAF	*p* ^c^		
Overall												
SUD	448	0.49	316	118	14	< **0.001**	750	146	0.163	< **0.001**	1.62 (1.26–2.09)	0.97
CTL	361	0.77	210	129	22		549	173	0.240			
Sex and age matched^f^												
SUD	290	0.38	208	73	9	**0.001**	489	91	0.157	**0.001**	1.70 (1.25–2.30)	0.94
CTL	290	0.87	168	105	17		441	139	0.24			
Male												
SUD	299	0.39	212	77	10	**0.041**	501	97	0.162	**0.041**	1.43 (1.00–2.05)	0.53
CTL	161	1.00	98	56	7		252	70	0.217			
Female												
SUD	149	1.00	104	41	4	**0.005**	249	49	0.164	**0.005**	1.76 (1.19–2.63)	0.85
CTL	200	0.58	112	73	15		297	103	0.258			
Age < 40												
SUD	116	0.55	77	34	5	**0.021**	188	44	0.190	**0.021**	1.72 (1.07–2.77)	0.67
CTL	101	1.00	51	42	8		144	58	0.287			
Age ≧ 40												
SUD	319	0.26	236	74	9	**0.003**	546	92	0.144	**0.003**	1.64 (1.19–2.25)	0.89
CTL	254	0.46	158	82	14		398	110	0.217			
Violent methods												
SUD	414	0.28	293	107	14	< **0.001**	693	135	0.163	< **0.001**	1.62 (1.25–2.10)	0.96
CTL	361	0.77	210	129	22		549	173	0.240			
SUD without possible or diagnosed psychiatric disease												
SUD	165	0.54	120	40	5	**0.003**	280	50		**0.003**	1.76 (1.24–2.55)	0.92
CTL	361	0.77	210	129	22		549	173	0.240			

*Note:* *p* < 0.05 is shown in bold.

Abbreviations: 5‐HTTLPR, serotonin‐transporter‐linked promoter region; CI, Confidence interval; CTL, controls; HWE, Hardy–Weinberg equilibrium; L, long allele; MAF, minor allele frequency; OR, odds ratio; S, short allele; SUD, sucuide decedents.

^a^ *p*‐values for Hardy–Weinberg equilibrium calculated with Haldane's exact test.

^b^ Genotypic *p*‐values were calculated with the Cochran–Armitage test (Benjamini–Hochberg adjustment).

^c^ Allelic *p*‐values were calculated with Fisher's exact test (Benjamini–Hochberg adjustment).

^d^ Odds ratio for suicide decedents on S allele.

^e^ Statistical power was estimated based on Cohen's h.

^f^ We matched sex and age of controls and suicide decedents with EZR (R version 4.2.2).

### 3.2 Logistic Regression Analysis

Logistic regression analysis adjusting for age and sex demonstrated that the 5‐HTTLPR genotype remained significantly associated with suicide death (OR = 1.59, 95% CI = 1.23–2.05, *p* < 0.001). In the overall cohort, a significant association was observed between sex and genotype. In contrast, in the age‐ and sex‐matched cohort, no significant association was found between sex and genotype, while the association between suicide death and genotype remained statistically significant (Table 3).

**TABLE 3:** Logistic regression analysis genotype of 5‐HTTLPR on suicide decedents, adjusting for sex and age.

Variable	Odds ratio	Confidence interval	Standard error	*p*
Overall				
Genotype	1.59	1.23–2.05	0.130	**< 0.001**
Sex	0.41	0.30–0.54	0.149	**< 0.001**
Age	0.99	0.98–1.00	0.004	0.063
Sex and age matched^a^				
Genotype	1.68	1.25–2.25	0.150	**< 0.001**
Sex	1.03	0.74–1.43	0.170	0.868
Age	1.00	0.99–1.01	0.004	0.793

*Note:* The regression formula is modeled as follow: phenotype (suicide decedents or control) ~ genotype of 5‐HTTLPR (SS, SL, or LL) + age + sex. Dummy variables were assigned as follows: For genotype, SS = 2, SL = 1, and LL = 0. For sex, female = 1 and male = 0.

Abbreviations: 5‐HTTLPR, serotonin‐transporter‐linked promoter region; L, long allele; S, short allele.

^a^ We matched the sex and age of controls and suicide decedents with EZR (R version 4.2.2).

### 3.3 Meta‐Analysis

**FIGURE 2:**
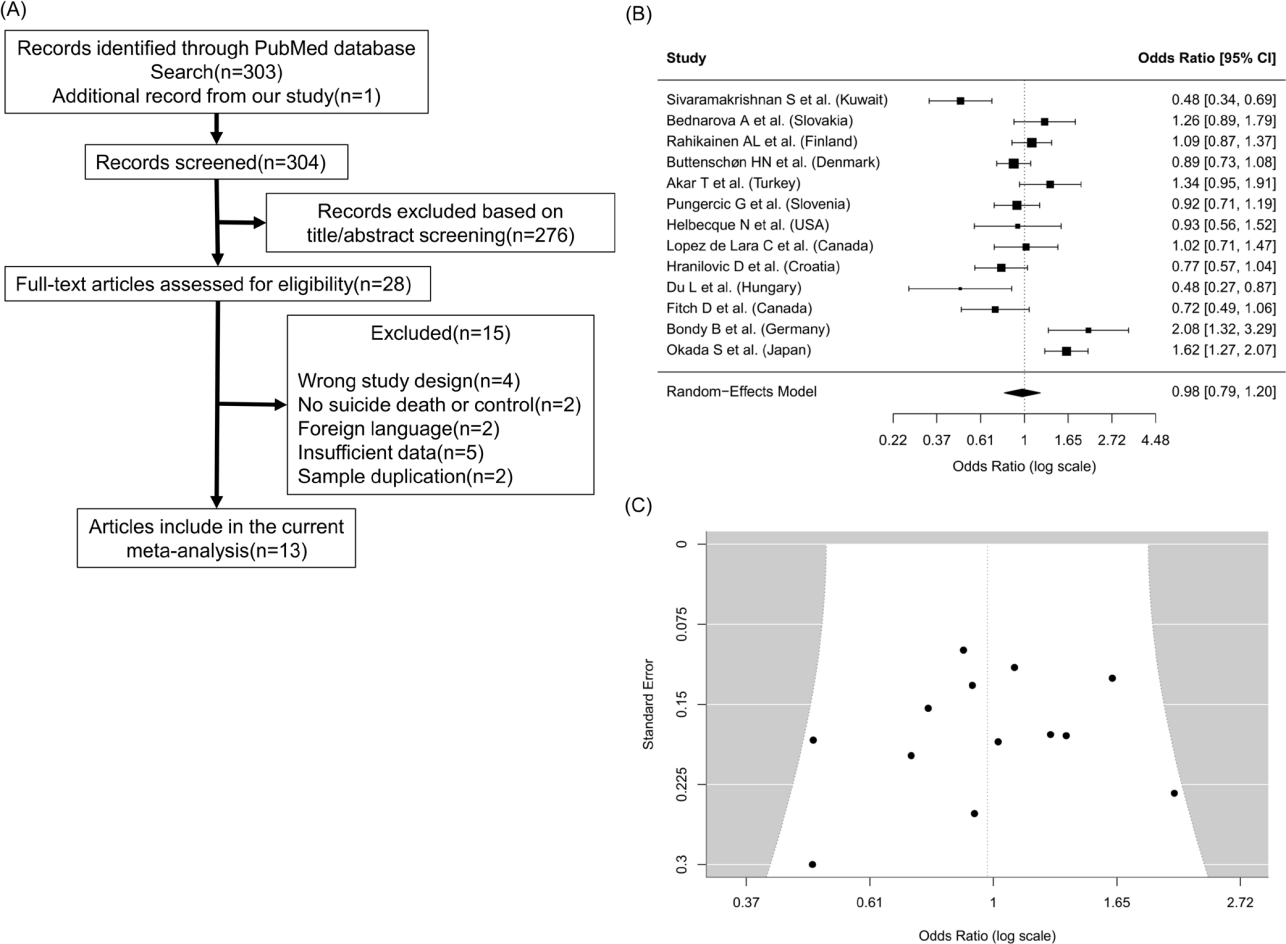
Overview and forest plot of the meta‐analysis of the 5‐HTTLPR polymorphism and suicide death. (A) The overview of this meta‐analysis. (B) The forest plot of this meta‐analysis. The horizontal axis shows the odds ratios and 95% confidence intervals of the short allele on suicide death. The vertical axis lists the first author of each study and the country where the samples were collected. The meta‐analysis was conducted using the R package metafor with a random‐effect model. (C) The funnel plot of this meta‐analysis.

A total of 13 studies, including the present dataset, met the criteria for inclusion in this meta‐analysis. An overview and the corresponding forest plot are shown in Figure 2. In contrast to our cohort findings, the meta‐analysis did not identify a significant association between 5‐HTTLPR alleles and suicide (OR = 1.00, 95% CI = 0.92–1.09; *p* = 0.989; *I* ^2^ = 79.66%). Visual inspection of the funnel plot and Egger's regression test based on the random‐effects model showed no evidence of funnel plot asymmetry (*z* = −0.81, *p* = 0.42).

## 4 Discussion

In this study, we observed a significant association between the S allele of 5‐HTTLPR polymorphism and suicide death in a Japanese cohort. In contrast, our meta‐analysis found differences between populations and did not show a significant association. The substantial heterogeneity observed in the meta‐analysis (*I* ^2^ ≈80%) indicates that the pooled estimate should be interpreted with caution. Such heterogeneity suggests the presence of meaningful between‐study differences, including ethnic composition, phenotype definition, and methodological variation. Therefore, the discrepancy between our Japanese cohort and the overall meta‐analytic result does not necessarily indicate inconsistency but rather highlights potential population‐specific effects that warrant further investigation. Ethnic differences in genetic architecture are well recognized in psychiatric genetics [18], and Japan—being an island population—may possess distinct genetic characteristics even within Asian countries [19]. Consistent with this, population differences in the allele frequencies of serotonin‐related polymorphisms have been reported [20]. Moreover, even for nominally identical polymorphisms, population‐specific variation in linkage disequilibrium structure and in the regulatory or enhancer elements surrounding these loci can substantially modify their functional effects on gene expression [21, 22]. Furthermore, given the polygenic nature of suicidal behavior [23, 24, 25], the influence of other genetic factors may obscure the detectable effects of individual variants such as SLC6A4 polymorphisms. To our knowledge, this study is the first to examine the association between 5‐HTTLPR and suicide death in a Japanese population and represents the largest analysis conducted in Asian samples to date. While these findings warrant cautious interpretation, they may suggest that 5‐HTTLPR variation could contribute to population‐specific genetic susceptibility to suicide in Japan.

Multiple lines of evidence support an important role of the serotonergic (5‐HT) system in suicidal behavior [26]. As a key monoaminergic neurotransmitter, 5‐HT is involved in numerous physiological functions and influences sensory processing, executive control, emotion regulation, learning, and memory [27]. Environmental stress has been shown to interact with 5‐HTTLPR variants, increasing vulnerability to mood disorders and suicidal behavior [28]. Behaviorally, the short allele has been suggested to be associated with increased stress sensitivity, heightened emotional reactivity, and greater impulsivity. These intermediate phenotypes may represent plausible pathways through which serotonergic genetic variation contributes to suicide vulnerability, particularly under environmental stress. Notably, the association remained significant even when individuals without diagnosed or suspected psychiatric disorders were analyzed separately (Table 2), suggesting that the observed genetic effect may not be solely mediated through major psychiatric illness. Suicidal behavior has also been linked to neural circuits governing emotional regulation and impulsivity [29]. These circuits include the prefrontal cortex, orbitofrontal cortex, and amygdala, each of which is modulated by serotonergic signaling [30, 31]. Specific 5‐HT receptor (5HTR) subtypes such as 5HTR2A and 5HT2C have been implicated in suicide risk [25]. For instance, psilocybin, the psychoactive compound in hallucinogenic mushrooms, binds to these receptor subtypes and modulates serotonergic neurocircuits and calcium signaling pathways [32, 33, 34].

Meta‐analytic evidence has indicated interactions between stressful life events, 5‐HTTLPR polymorphisms, and depressive severity [35]. The serotonin transporter gene has been widely studied in psychiatric genetics, with findings highlighting complex interactions with clinical phenotypes [36]. Epigenetic regulation—such as DNA methylation of the SLC6A4 promoter—has also been implicated in depression and stress‐related psychopathology [37]. Pharmacogenetic studies further suggest that 5‐HTTLPR may influence treatment response to antidepressants [38].

The 5‐HTT binding site is located on serotonergic terminals and platelets, reflecting transporter density and neural integrity [39]. The SS and SL genotypes of 5‐HTTLPR have been associated with approximately 40% fewer binding sites compared with the LL genotype [25]. Earlier candidate gene studies reported possible associations between 5‐HTTLPR and mood‐related phenotypes [40]. However, large‐scale meta‐analyses have failed to support a robust main effect of this polymorphism on depression risk [41, 42]. These findings suggest that suicidal behavior may not be explained solely by depressive pathology and likely reflect a more complex interplay of genetic and behavioral factors. Suicide risk cannot be explained solely by depression; traits such as impulsivity, aggression, and substance misuse contribute independently and may represent intermediate phenotypes relevant to suicidality [43]. Incorporating these behavioral and neurobiological factors into future studies may help clarify how serotonergic genetic variation influences suicide vulnerability.

This study has several limitations. *First*, the primary case–control analysis was conducted in a cohort restricted to individuals of Japanese ancestry. While the subsequent meta‐analysis incorporated data from cohorts of diverse ethnic backgrounds, the findings from the Japanese sample may not be directly generalizable to other populations. *Second*, we focused exclusively on the 5‐HTTLPR polymorphism and were unable to assess other genetic variants that may influence *SLC6A4* expression or serotonergic signaling pathways. *Third*, although subgroup analyses and covariate‐adjusted logistic regression models were performed, baseline differences in sex and age between suicide decedents and control subjects may have partially influenced the observed associations. Furthermore, control participants were rigorously screened to exclude any personal or family history of psychiatric disorders. This stringent selection may have resulted in a “super‐healthy” control group that is not fully representative of the general population. Such selection bias could potentially inflate the observed effect size and should be considered when interpreting the findings. Additionally, the present study employed a biallelic (L/S) classification of 5‐HTTLPR and did not distinguish the triallelic variants (LA and LG). Prior functional work suggests that the LG allele shows transcriptional activity similar to the S allele; therefore, potential functional misclassification within the L allele cannot be excluded [16]. Importantly, studies in Japanese populations have reported a complex allelic architecture and population‐specific allele frequencies of 5‐HTTLPR, indicating that variation within the “L” category (including low‐activity alleles) may be relevant for functional interpretation [20, 44]. This limitation may affect functional interpretation of our findings, and future studies incorporating triallelic genotyping (including rs25531 where feasible) are warranted. In addition, the number of individuals using non‐violent suicide methods was relatively small, which limited statistical power for method‐specific comparisons.

## 5 Conclusion

In conclusion, the 5‐HTTLPR S allele may be associated with suicide death in a Japanese cohort population. Studies of this association in Asian populations remain limited, and our dataset represents the largest investigated to date. While replication in diverse and larger samples is necessary, these findings may provide insight into population‐specific genetic factors underlying vulnerability to suicide.

## Author Contributions

**Shohei Okada:** conceptualization; methodology; formal analysis; investigation; data curation; visualization; writing‐original draft preparation. **Toshiyuki Shirai:** formal analysis; data curation; visualization; funding acquisition; writing‐original draft preparation. **Masao Miyachi:** methodology; formal analysis; investigation; data curation. **Gentaro Yamasaki:** data curation; resources. **Kiriko Minami:** data curation. **Ryo Tsukamoto:** data curation. **Kentaro Mouri:** data curation. **Takaki Tanifuji:** methodology; data curation. **Ryuichi Katada:** data curation; resources. **Satoshi Okazaki:** conceptualization; methodology; funding acquisition; data curation. **Ikuo Otsuka:** conceptualization; methodology; funding acquisition; data curation. **Akitoyo Hishimoto:** conceptualization; funding acquisition; writing – review and editing; supervision; project administration.

## Funding

This work was partially supported by JST Moonshot R&D Program (Grant Number JPMJMS239F to Akitoyo Hishimoto and Ikuo Otsuka), JSPS KAKENHI (Grant Numbers JP25K19058 to Toshiyuki Shirai; JP21K07520, JP24K10710 to Satoshi Okazaki; JP24K10710, JP24K10732 to Ikuo Otsuka; JP21H02852, JP24K02383 to Akitoyo Hishimoto), Japan Agency for Medical Research and Development grants (Grant Number 23dk0307111 to Ikuo Otsuka and Akitoyo Hishimoto), SENSHIN Medical Research Foundation (to Ikuo Otsuka and Takaki Tanifuji), and the Smoking Research Foundation (to Akitoyo Hishimoto and Ikuo Otsuka).

## Disclosure

The authors have nothing to report.

## Ethics Statement

This study was approved by the Ethics Committee of the Graduate School of Medicine, Kobe University. In accordance with the Declaration of Helsinki, all controls and families of suicide decedents were given sufficient written and verbal explanations about the study and provided their informed consent.

## Consent

All participants (controls and families of suicide decedents) gave their consent to participate in this study both verbally and in writing and for publication of this study.

## Conflicts of Interest

The authors declare no conflicts of interest.

## Supporting information


**Table S1:** Provides participant‐level data used in this study.

## Data Availability

The data that supports the findings of this study are available in the supporting information of this article. Detailed data for each participant are provided in Table S1.
